# Characterization of multidrug-resistant potential pathogens isolated from milk and some dairy products in Egypt

**DOI:** 10.5455/javar.2023.j679

**Published:** 2023-06-30

**Authors:** Dina Ashraf, Rabee A. Ombarak, Ahmed Samir, Ayah B. Abdel-Salam

**Affiliations:** 1 Department of Microbiology, Faculty of Veterinary Medicine, Cairo University, Cairo, Egypt; 2 Department of Food Hygiene and Control, Faculty of Veterinary Medicine, University of Sadat City, Sadat, Egypt; 3 Department of Food Hygiene and Control, Faculty of Veterinary Medicine, Cairo University, Cairo, Egypt

**Keywords:** Antimicrobial resistance, dairy products, potential pathogens, public health

## Abstract

**Objective:**

This study aimed to explore the incidence and antimicrobial resistance (AMR) of *Escherichia coli*,* Staphylococcus aureus*, and *Bacillus cereus *in raw milk and some Egyptian dairy products, namely Kariesh cheese and rice with milk.

**Material and Methods:**

112 samples (70 raw milk, 30 Kariesh cheese, and 12 rice with milk) were randomly collected from different districts in Cairo and Giza, Egypt. The samples were examined for *E. coli*,* S. aureus*, and* B. cereus *presence. The susceptibility of the obtained isolates was tested against 11 antimicrobials using the disk diffusion method, and further, the presence of AMR genes was examined.

**Results:**

The incidences of *E. coli*,* S. aureus*, and* B. cereus *were 69.64%, 12.5%, and 16.7% in the examined samples, respectively*. *The antibiogram indicated that *E. coli *isolates (*n =* 60) were resistant to gentamycin (73.33%), ampicillin (AM, 53.3%), and cefotaxime (CTX, 16.66%). Multidrug-resistant (MDR) *E. coli *strains (*n =* 5) were tested for β-lactams resistance genes. *bla*_TEM_ was detected in all isolates, and two of them additionally carried *bla*_CTX-M_. *Staphylococcus aureus* isolates (*n =* 10) were resistant to AM (100%), followed by tetracycline (TE), CTX, and gentamycin (60% each). All MDR *S. aureus* strains (*n =* 4) carried *bla*Z and *tetK*, and three of them additionally carried *aac*(6’)-*aph* (2’’). *Bacillus cereus* isolates (*n =* 30) showed resistance to AM (100%), amoxicillin (20%), and TE (6.66%). *bla* and *tet*A genes were detected in all MDR *B. cereus* isolates (*n =* 6).

**Conclusion:**

Our findings denote the high incidence of potential health hazards in raw milk and some of its products and the existence of AMR bacteria, including MDR strains, which can cause human illnesses that are difficult to treat.

## Introduction

Milk is the most complete food, as it is a good source of many essential nutrients that make it one of the most fundamental foods for all age categories and plays a key role in the diet of over 6 billion people in the world [1]. On the other side, it provides favorable environmental conditions for microbial growth, especially for pathogenic bacterial species [2].

Several bacterial pathogens, such as *Staphylococcus aureus*,* Bacillus cereus*, and *Escherichia coli*, were isolated from raw milk and dairy products [3]. The occurrence of such pathogens in milk constitutes a public health threat, specifically among individuals who have weakened immune systems or those who consume unpasteurized raw milk or its products [4].

Bacterial contamination of milk with pathogenic microorganisms may result from many factors, including the milking process, utensils, environment, and personnel. In addition, it may be contaminated during unhygienic storage, and transportation [5].

The presence of enteric bacteria, including *E. coli*, in food is a reliable indicator of fecal contamination [6]. Although most *E. coli* present as commensals, many are opportunistic pathogens that can cause gastrointestinal illness and can be used as a bio-indicator of antimicrobial resistance (AMR) [7].

*Staphylococcus aureus* is categorized as the third causal agent of foodborne diseases worldwide. It is considered one of the most important foodborne pathogens isolated from milk and dairy products. Its isolation is an indicator of neglected hygienic measures employed during the production, handling, and distribution of milk and dairy products or contamination due to mastitis or contaminated food handlers [8].

*Bacillus cereus* is a major foodborne pathogen that has a bad impact on heat-treated milk as its thermophilic endospores can withstand the pasteurization process and can germinate and produce spoilage enzymes, leading to off flavors in the pasteurized milk. Ingestion of food contaminated with *B. cereus *or its toxins can cause severe gastrointestinal illness with diarrhea and without significant upper intestinal symptoms, which is the commonly known manifestation of the disease [9].

Multidrug-resistant (MDR) microorganisms have become a great threat to public health worldwide [3]. The uncontrolled use of antibiotics, in therapeutic and sub-therapeutic points, in dairy cows increases the incidence of MDR pathogens in raw milk and the subsequent incidence in its products [10].

The antibiotic-resistant pathogens could transmit from animals to humans in different ways, including through the ingestion of contaminated milk and dairy products directly or through cross-contamination [11]. The AMR genes spread among microbes in the dairy environment pose a risk that may come into contact with humans through the processing steps or consumption of contaminated dairy products [12].

Raw milk and products made from it are considered one of the focal sources for outbreaks with antibiotic-resistant pathogens in developing countries, because of the presence of several contamination sources due to poor hygienic practices, inadequate regulations concerning food safety, insufficient resources, neglected food management systems, and bad personal hygiene by handlers [3]. Therefore, together with investigating the incidence rates, investigating the AMR resistance phenotypes of the pathogens obtained from food sources is crucial. Furthermore, the available data about AMR prevalence and its molecular basis in bacteria from Egyptian food is still scarce. Therefore, the purpose of the current study was to investigate the incidence of some pathogens such as *E. coli*,* S. aureus*, and *B. cereus* in raw milk and some Egyptian dairy products, namely Kariesh cheese and rice with milk, and examine the AMR of the isolated bacteria.

## Material and Methods

### Study samples

A total of 112 samples, including 70 samples of raw milk, 30 Kariesh cheese (Egyptian soft cheese) samples, and 12 rice with milk (a traditional Egyptian dessert). Samples were randomly collected in their retail containers during the period from December 2020 to April 2021 at the consumer level from street vendors, grocery stores, and dairy shops at different markets and from dairy farms in Cairo and Giza governorates, Egypt. The samples were collected and transported to the laboratory in an insulated icebox with the minimum delay.

### Sample preparation, isolation, and identification of pathogenic bacteria (E. coli, S. aureus, and B. cereus)

Twenty-five milliliters of raw milk or 25 gm of the other dairy products were prepared according to International Organization for Standardization (ISO) 6887-5 [13]. All samples were examined for the incidence of *E. coli*,* S. aureus*, and* B. cereus* as described previously [14–16]. Biochemical identification of the obtained isolates was done according to the methods recommended by APHA [17,18] and ISO 7932 [16].

### Antimicrobial susceptibility testing

Antimicrobial susceptibility patterns of the isolates were determined using the Kirby–Bauer disk diffusion method, and the results were interpreted according to CLSI guidelines [19]*.*

*Escherichia coli *isolates were tested against eight commercially available antimicrobial disks: ampicillin (AM, 10 μg), amoxicillin-clavulanic acid (AMC, 30 μg), cefotaxime (CTX, 30 μg), ceftazidime (CAZ, 30 μg), chloramphenicol (C, 30 μg), ciprofloxacin (CIP, 5 μg), tetracycline (TE, 30 μg), and trimethoprim–sulfamethoxazole (SXT-25 μg).

For testing *S. aureus *isolates, eight antibiotics that are frequently used in veterinary and human illnesses were selected, such as AM (10 μg), AMC (30 μg), CTX (30 μg), CIP (5 μg), Gentamicin (GM, 10 μg), TE (30 μg), SXT (25 μg), and Vancomycin (VA, 30 μg). *Bacillus cereus *isolates were tested against amoxicillin (AX, 25 μg), AM (10 μg), TE (30 μg), and VA (30 μg) (Oxoid, UK).

### Detection of AMR genes

*Escherichia coli*,* S. aureus*, and* B. cereus* isolates that exhibited MDR phenotypes were examined for the presence of AMR genes relevant to each main phenotype. The presence of genes linked with β-lactam resistance [*bla*_TEM_,* bla*_CTX-M_ for *E. coli*; *blaZ* for *S. aureus* and *bla* for *B. cereus*], TE resistance [*tetK *in *S. aureus *and *tetA* in *B. cereus*], and aminoglycosides [*aac*(*6’*)*aph *(*2’’*) in *S. aureus*] were detected using polymerase chain reaction (PCR) as previously described [20].

The predicted sizes of PCR products for different AMR genes and primer sequences used for their detection are mentioned in Table 1. The PCR products were visualized under UV light (Alpha Innotech, San Leandro, USA) after electrophoresis using 1.5% agarose gels (ABgene, Surrey, UK).

**Table 1.** Primers for the detection of antimicrobial-resistant genes and for the identification of gene cassettes.

Reference	Amplicon size	Primer sequence (5’–3’)	Gene	Target
[21]	593 bp	ATGTGCAGYACCAGTAARGTKATGGC	*bla*_CTX-M_	*E. coli*
		TGGGTRAARTARGTSACCAGAAYCAGCGG		
[22]	516 bp	ATCAGCAATAAACCAGC	*bla*_TEM_	
		CCCCGAAGAACGTTTTC		
[23]	833 bp	TACAACTGTAATATCGGAGGG	*blaZ*	*S. aureus*
		CATTACACTCTTGGCGGTTTC		
[24]	360 bp	GTAGCGACAATAGGTAATAGT	*tetK*	
		GTAGTGACAATAAACCTCCTA		
	491 bp	GAAGTACGCAGAAGAGA	*aac*(*6’*)*aph *(*2’’*)	
		ACATGGCAAGCTCTAGGA		
[25]	502 bp	GGCGGTCTTCTTCATCATGC	*tetA*	*B. cereus*
		CGGCAGGCAGAGCAAGTAGA		
[26]	680 bp	CATTGCAAGTTGAAGCGAAA	*bla*	
		TGTCCCGTAACTTCCAGCTC		

## Results

### Incidence of pathogenic bacteria (E. coli, S. aureus, and B. cereus) in the examined samples

The results of bacterial isolation from 112 samples (70 raw milk, 30 Kariesh cheese, and 12 rice with milk) are represented in Table 2. Out of the 112 tested samples, *E. coli *was isolated from 58 (82.85%), 16 (53.33%), and 4 (33.33%) raw milk, Kariesh cheese, and rice with milk samples, respectively, with an overall isolation percentage of 78 (69.64%).

*Staphylococcus aureus *incidence in the three types of tested samples is shown in Table 2. Twelve raw milk samples (17.14%) and two (6.66%) of the cheese samples were contaminated with *S. aureus*, while all rice with milk samples (*n =* 12) were not contaminated. The overall occurrence of *S. aureus *in the different types of samples was 12.5%.

Concerning the *B. cereus* isolation rate, Table 2 shows that the highest incidence was in rice with milk samples (66.66%), while for raw milk, eight samples were positive with an incidence of 11.42%), and only two samples (6.66%) from Kariesh cheese were positive for *B. cereus*.

### AMR phenotypes and genotypes of E. coli, S. aureus, and B. cereus isolates

The antibiogram of the isolates is displayed in Table 3. The results indicated a higher resistance rate of tested *E. coli *isolates (60 isolates) against gentamycin, where 44 (73.33%) isolates were resistant, followed by AM, for which 32 (53.3%) isolates were resistant, and 10 (16.66) were resistant to CTX. However, all isolates showed sensitivity to TE, CIP, and trimethoprim–sulfamethoxazole. Concerning *S. aureus*, all examined isolates (10 isolates) were resistant to AM, and 6 (60%) isolates were resistant to TE, CTX, and gentamycin. On the other hand, all isolates were sensitive to CIP, AMC acid, and trimethoprim–sulfamethoxazole. Of the 30 *B. cereus *tested isolates, all were resistant to AM, followed by AX 6 (20%) and TE 2 (6.66). All isolates were sensitive to VA.

Results in Table 4 illustrate the incidence of MDR strains among the recovered isolates. The highest MDR rate was met with *S. aureus*, of which 4 out of 10 *S. aureus* isolates (40%) conferred resistance to 3 antimicrobials belonging to different categories in 2 isolates, and the other 2 were resistant to 4 agents. After which, 6 isolates out of 30 (20%) of *B. cereus *showed an MDR pattern. On the other hand, 5 out of 60 *E. coli *isolates (8.33%), all of which were isolated from raw milk, showed an MDR profile.

**Figure 1.**
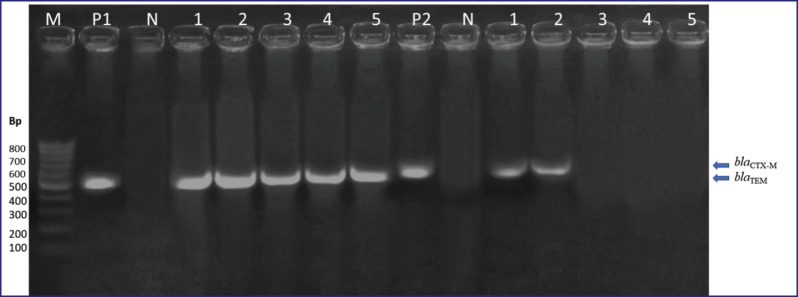
β-lactams resistance genes (*bla*_TEM_, *bla*_CTX-M_) in MDR *E. coli *isolates. Lanes: M, marker, 100-bp ladder; P1, positive control for *bla*_TEM_; P2, positive control for *bla*_CTX-M_; N, negative control DW; 1–5, examined *E. coli *isolates.

**Figure 2.**
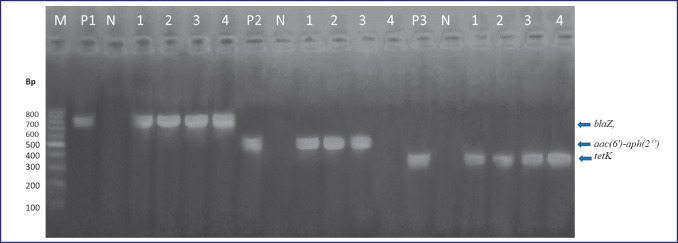
*blaZ*,* aac*(*6’*)*-aph *(*2’’*) and* tetK *genes in MDR *S. aureus* isolates. Lanes: M, marker, 100-bp ladder; P1, positive control for *blaZ*; P2, positive control for *aac*(*6’*)*-aph*(*2’’*); P3, positive control for *tetK*; N, negative control DW; 1–4, examined *S. aureus* isolates.

**Figure 3.**
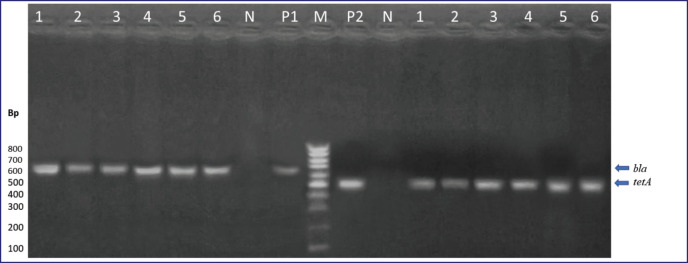
*bla *and* tetA *genes in MDR *B. cereus *isolates. Lanes: M, marker, 100-bp ladder; P1, positive control for *bla*; P2, positive control for *tetA*; N, negative control DW; 1–6, examined *B. cereus *isolates.

As represented in Table 5, five *E. coli* isolates with MDR patterns were subjected to genotyping; all five isolates had *bla*_TEM_ genes, and 40% of them were positive for *bla*_CTX-M_. The presence of the* bla*Z gene and *tet*K were confirmed in all MDR isolates of *S. aureus* (four isolates) with 100% incidence, and the *aac*(*6’*)*aph *(*2’’*) gene was positive in three (75%) isolates. These three genes are the most involved in the antibiotic resistance of *S. aureus* strains. For *B. cereus*, all six isolates possessed the *bla *and *tet*A genes (100%). The resistance gene PCR product patterns are shown in Figures 1–3.

## Discussion

Milk and its products are vital sources of food for the human population all over the world. Despite their numerous health benefits, milk is an optimum medium for numerous bacteria that could represent human health hazards, such as *E. coli*,* S. aureus*, and* B. cereus*, which are involved in most foodborne illnesses. In addition to the danger of food poisoning, milk and its products may be potential sources of MDR bacterial strains due to the misuse of antibacterial therapeutics in dairy farm management. These MDR strains constitute a huge complication for consumers at the level of antibacterial therapeutic efficiency. The MDR determinants of transmission to other bacterial pathogens have clinical significance [20].

**Table 2.** Incidence of pathogenic bacteria in examined raw milk and dairy products samples.

Sample type	No. of samples	*E. coli*		*S. aureus*		*B. cereus*	
		No. of positive samples (%)	No. of isolates	No. of positive samples (%)	No. of isolates	No. of positive samples (%)	No. of isolates
Raw milk	70	58 (82.85%)	60	12(17.14%)	16	8 (11.42%)	14
Kariesh cheese	30	16 (53.33%)	16	2 (6.66%)	6	2 (6.66%)	6
Rice with milk	12	4 (33.33%)	6	0 (0%)	0	8 (66.66%)	24
Total	112	78 (69.64%)	82	14 (12.5%)	22	18 (16.07%)	44

**Table 3.** Antibiotics sensitivity of isolated pathogens (*E. coli*,* S. aureus*, and* B. *cereus) from examined raw milk and dairy product samples.

Antimicrobial agent^a^	*E. coli* No. (%) of isolates (*N* = 60)			*S. aureus* No. (%) of isolates (*N* = 10)			*B. cereus* No. (%) of isolates (*N* = 30)		
	R (%)	I (%)	S (%)	R (%)	I (%)	S (%)	R (%)	I (%)	S (%)
AM	32 (53.3)	24 (40)	4 (6.66)	10 (100)	0 (0)	0 (0)	30 (100)	0 (0)	0 (0)
AMC	2 (3.33)	16 (26.66)	42 (70)	0 (0)	0 (0)	10 (100)	NA	NA	NA
GM	44 (73.33)	14 (23.33)	2 (3.33)	6 (60)	4 (40)	0 (0)	NA	NA	NA
TE	0 (0)	0 (0)	60 (100)	6 (60)	0 (0)	4 (40)	2 (6.66)	0 (0)	28 (93.33)
CTX	10 (16.66)	0 (0)	50 (83.33)	6 (60)	0 (0)	4 (40)	NA	NA	NA
CAZ	8 (13.33)	0 (0)	52 (86.66)	NA	NA	NA	NA	NA	NA
C	0 (0)	10 (16.66)	50 (83.33)	0 (0)	2 (20)	8 (80)	NA	NA	NA
CIP	0 (0)	0 (0)	60 (100)	0 (0)	0 (0)	10 (100)	NA^b^	NA	NA
SXT	0 (0)	0 (0)	60 (100)	0 (0)	0 (0)	10 (100)	NA	NA	NA
AX	NA	NA	NA	NA	NA	NA	6 (20)	18 (60)	6 (20)
VA	NA	NA	NA	NA	NA	NA	0 (0)	0 (0)	30 (100)

a AM, ampicillin (10 μg); AMC, amoxicillin-Clavulanic A, (30 μg); AX, amoxicillin (25 μg); C, chloramphenicol (30 μg); CAZ, ceftazidime (30 μg); CIP, ciprofloxacin (5 μg); CTX, cefotaxime (30 μg); GM, gentamycin (10 μg); SXT, trimethoprim–sulfamethoxazole (25 μg); TE, tetracycline (30 μg); VA, vancomycin (30 μg).

b NA, Not applicable.

**Table 4.** MDR isolates of *E. coli*,* S. aureus*, and* B. cereus* isolated from examined raw milk and dairy product samples.

Isolated pathogen	No. of isolates with MDR pattern	MDR patterns^a^
*E. coli* (*n =* 5)	3 2	AM, CTX, CAZ CTX, GM, CAZ
*S. aureus *(*n =* 4)	2	AM, GM, TE
	2	AM, CTX, GM, TE
*B. cereus *(*n =* 6)	6	AM, AX, TE

a AM, ampicillin (10 μg); AX, amoxicillin (25 μg); CAZ, ceftazidime (30 μg); CTX, cefotaxime (30 μg); GM, gentamycin (10 μg); TE, tetracycline (30 μg).

**Table 5.** Distribution of resistance genes in the selected MDR isolates of *E. coli*,* S. aureus*, and* B. cereus* isolated from examined raw milk and dairy products samples.

Target genes	*E. coli* (*n*^a^ = 5)	*S. aureus* (*n* = 4)	*B. cereus* (*n* = 6)
*bla*_TEM_	5 (100%)	-	-
*bla*_CTX-M_	2 (40%)	-	-
*blaZ*	-	4 (100%)	-
*aac*(*6’*)*aph *(*2’’*)	-	3 (75%)	-
*tetK*	-	4 (100%)	-
*bla*	-	-	6 (100%)
*tetA*	-	-	6 (100%)

a *n =* total number of isolates.

The Egyptian standard (ES) ES:154-1 [27] asserts that raw milk must be free from pathogenic bacteria and their toxins. The results of the current study highlighted that *E. coli *was the most prevalent among the investigated pathogens in the examined milk and cheese samples, which could be due to the wide spreading of *E. coli *on the animal body and in the environment as the organism is a common fecal resident. Out of 112 tested samples, *E. coli *was isolated from 58 (82.85%), 16 (53.33%), and 4 (33.33%) from raw milk, Kariesh cheese, and rice with milk samples, respectively, with an overall isolation percentage of 78 (69.64%) (Table 2). The occurrence of *E. coli* in raw milk and its products is an indicator of poor hygienic measures during the milking process, insufficient cleaning and sanitation of dairy utensils, worker hands, animal udders, or post-process contamination. The findings of Sultana et al. [28], who isolated *E. coli* from yogurt samples, support the isolation of *E. coli* from rice with milk despite being heat treated, which may result in post-process contamination.

The high incidence of *E. coli *in milk and cheese samples was previously reported by Selvamalar et al. [29] and by Ibrahim et al. [30], who stated 52% and 48% prevalence rates of *E. coli*, respectively, in raw milk in Sudan and Pakistan. On the other hand, the prevalence of *E. coli *was high in other reports, but with lesser rates than ours (26% by Sultana et al. [28], 24% by Selvamalar et al. [29]), while other studies failed to isolate *E. coli* from raw milk and cheese samples as mentioned by Awaad et al. [31]. This variation in the incidence rates of *E. coli* could be due to differences in the origin of samples, the techniques used for their collection and transportation, or environmental conditions.

Considering the incidence of *S. aureus *in the examined samples as tabulated in Table 2,* S. aureus *was isolated from raw milk at a higher rate (17.14%) than from Kariesh cheese (6.66%), while it failed to be detected in rice with milk samples. The overall incidence of *S. aureus *in the different types of samples was 12.5%. These results may be attributed to the contamination of raw milk during production, which could be easily controlled during dairy product processing either via a high acidity percentage as in the case of Kariesh cheese or via heat treatment as in the case of desserts. Zeinhom et al. [32] indicated that the high incidence of *S. aureus* in raw milk samples from Beni Suef, Egypt, was due to contamination from the environment, cross-contamination, and poor handling in milk collection centers or during transportation. In addition, the udders of infected animals were also blamed. Also, Awaad et al. [31] isolated *S. aureus *from raw milk produced in Fayoum farms, Egypt, before cheese production, but with a higher incidence level (40%), this incidence was decreased to 8% after ripening of cheese produced from the same milk, indicating the effect of ripening changes on *S. aureus* survival.

According to the ES:154-1 [27], for raw milk, the *S. aureus* count must not be more than 1 × 10^2^ colony forming unit/ml, while for other dairy products such as Kariesh cheese, it must be free from it or its toxins. However, in the current study, 6.7% of Kariesh cheese samples were contaminated with the organism; Zeinhom et al. [32] isolated *S. aureus *from Kariesh cheese in Egypt with a higher incidence rate (18%). This variation in the results could be attributed to the fact that this product is usually produced on a small scale or homemade, in addition to the absence of a heating process or pasteurization of milk during its manufacturing and its low salt content.

Looking at the incidence of *B. cereus*, it was clear that the organism was more prevalent in the rice with milk than in other samples. Data presented in Table 2 showed that eight samples were positive from the raw milk samples, with an incidence of 11.42%. Only two samples from Kariesh cheese were positive for *B. cereus*, with an incidence percentage of 6.66%, while the highest incidence for *B. cereus* was in rice with milk samples (66.66%). *Bacillus cereus* contamination is related to the efficacy of the hygienic measures applied during the processing and distribution of milk products.

The low prevalence of *B. cereus* in Kariesh cheese samples examined in the current study comes in agreement with previous reports that indicated that the acidity of such kinds of cheese acts as a control measure [33]. On the contrary, the high prevalence of *B. cereus* in rice and milk samples could be due to the slight boiling during cooking, which leads to the destruction of most of the vegetative bacterial species, leaving the heat-resistant *B. cereus* spores to flourish after cooling.

Rice pudding milk is a popular dairy dessert in Egypt among people of different ages due to its pleasant and satiating power. However, its contamination with *B. cereus* is high due to its unhygienic processing, storage, and distribution. *Bacillus cereus* is one of the most isolated pathogens from this product [34].

Together with investigating the incidence rates, investigating the antibiotic resistance phenotypes of the pathogens isolated from food sources is crucial; therefore, it was determined for isolated pathogens in our study as shown in Table 3.

*Escherichia coli *isolates showed high resistance rates against GM (73.33%), AM (53.3%), and CTX (16.66%) and were moderately sensitive to chloramphenicol, CAZ, CTX, and AX with clavulanic acid, while all the isolates showed sensitivity to TE, trimethoprim–sulfamethoxazole, and CIP.

All isolates of *S. aureus* (100%) were resistant to AM, with TE, CTX, and gentamycin (60%) coming in second and third place, respectively. TEs are broad-spectrum antimicrobial agents often used in the treatment of infections in dairy animals. The high prevalence of resistance against TE has been reported previously among *E. coli *and* S. aureus* isolated from raw milk and dairy products from different countries [35]. On the other hand, all the isolates (100%) were sensitive to CIP, AX, clavulanic acid, and trimethoprim–sulfamethoxazole.

Regarding *B. cereus*, all isolates (100%) showed resistance to AM, followed by AX (20%) and TE (6.66%). All isolates (100%) were sensitive to VA.

Our findings coincide with some previous studies, such as Zeinhom et al.’s [32] study, in which *S. aureus* isolates from raw milk and dairy products such as cheese were resistant to AM (72%), and TE (60%).

MDR is defined as acquiring non-susceptibility to at least one agent in three or more antimicrobial classes [36]. Results in Table 4 illustrate the incidence of MDR strains among the recovered isolates. The highest MDR rate was met with *S. aureus*, of which 4 isolates out of 10 (40%) conferred resistance to 3 antimicrobials belonging to different categories, with 1 of them even resistant to 4 agents. On the other hand, only 5 out of 60 *E. coli* isolates (8.33%) and 6 out of 30 *B. cereus* isolates (20%) showed a MDR profile.

Concerning *E. coli*, the prevalence of MDR isolates detected in the present study is not as frightening as what was reported by Ombarak et al. [20], who reported that 50% of *E. coli *isolates recovered from raw milk and cheese samples in Egypt was MDR. The absolute resistance of *B. cereus *to AM agrees with the report of Osama et al. [33]. Detection of even one MDR isolate of *B. cereus*, as in the current study, is alarming as the bacterium is a spore-former and able to reside in the environment for long periods while carrying this transmissible criterion.

The high resistance levels, with a high proportion for beta-lactams, among the examined isolates obtained in this study may be due to the increased usage of beta-lactams in the veterinary field.

In this study, all the tested resistant isolates of *E. coli* were found to carry the* bla*_TEM_ gene (*n =* 5) at 100% (Table 5), which agrees with the previous findings [33] from food and food-producing animals. In the same context, Yu et al. [37] reported that 83.1% of *E. coli* isolates from raw milk samples had the *bla*_TEM_ gene, while 40% of these isolates carried the *bla*_CTX-M_ gene. Hassani et al. [3] found that 50% of *E. coli* isolates had *bla*_TEM_. The same genes were detected in resistant *E. coli* from dairy animals [38]. It was noted that all isolates possessing both genes showed resistance to AM, AX, and CAZ in phenotypic experiments.

All tested resistant *S. aureus* isolates (*n =* 4) possessed the *blaZ* and *TetK* genes, whereas the *aac6’-aph 2’’ *gene was found in only three isolates, as presented in Table 5. Ronco et al. [39] researched the prevalence of resistance genes in *S. aureus* isolates from dairy cows with clinical mastitis and bulk tank milk and detected *blaZ* in 17.2% of isolates. Liu et al. [40] stated in their study that 100% of *S. aureus* isolates carried the *aac6’-aph 2’’* gene, and the *tet* gene was detected in 14.3%.

The detection of MDR *B. cereus* isolates possessing the *bla* and *tet*A genes is worrisome, as the emergence of MDR pathogenic bacteria can be a serious hazard [41]. Ranjbar and Sami [42] highlighted the risk of different extended spectrum β-lactamases genes (antibiotic resistance genes) being transferred among bacteria in the environment, with the resultant threat to the efficacy of available antibiotics currently used in medical applications.

Further studies with a high number of samples and different dairy products are needed to elucidate the incidence of MDR pathogens in different dairy products produced from different locations in Egypt. Also, investigating the routes of raw milk contamination with AMR bacteria is important to uncover whether these bacteria access the milk from the animal or contaminate the milk during, after, or during processing. Moreover, an investigation of the antibiotic resistance of the MDR pathogens is required.

## Conclusion

The safety of raw milk and milk products investigated in the current study is not satisfactory as MDR-potential pathogens were detected. Sequentially, such products constitute a potential public health hazard. Moreover, the existence of MDR strains exaggerates the problem and calls for attention to the urgent need for decisive rules and regulations to face the increasing misuse of antibacterial therapeutics in dairy herd management and emphasize the need for new natural antimicrobial therapeutic agents.

## List of abbreviations

AMR: Antimicrobial resistance; APHA, American Public Health Association; CLSI, Clinical and Laboratory Standards Institute; ES, Egyptian Standards; ISO, International Organization for Standardization; MDR: Multi-drug resistant; PCR: Polymerase chain reaction.
